# V_H_‐V_L_ interdomain dynamics observed by computer simulations and NMR

**DOI:** 10.1002/prot.25872

**Published:** 2020-01-14

**Authors:** Monica L. Fernández‐Quintero, Valentin J. Hoerschinger, Leonida M. Lamp, Alexander Bujotzek, Guy Georges, Klaus R. Liedl

**Affiliations:** ^1^ Institute of General, Inorganic and Theoretical Chemistry, and Center for Molecular Biosciences Innsbruck (CMBI) University of Innsbruck Innrain Austria; ^2^ Roche Pharma Research and Early Development Large Molecule Research, Roche Innovation Center Munich Penzberg Germany

**Keywords:** antibodies, molecular dynamics simulations, NMR, V_H_ and V_L_ domain orientation

## Abstract

The relative orientation of the two variable domains, V_H_ and V_L_, influences the shape of the antigen binding site, that is, the paratope, and is essential to understand antigen specificity. ABangle characterizes the V_H_‐V_L_ orientation by using five angles and a distance and compares it to other known structures. Molecular dynamics simulations of antibody variable domains (Fvs) reveal fluctuations in the relative domain orientations. The observed dynamics between these domains are confirmed by NMR experiments on a single‐chain variable fragment antibody (scFv) in complex with IL‐1β and an antigen‐binding fragment (Fab). The variability of these relative domain orientations can be interpreted as a structural feature of antibodies, which increases the antibody repertoire significantly and can enlarge the number of possible binding partners substantially. The movements of the V_H_ and V_L_ domains are well sampled with molecular dynamics simulations and are in agreement with the NMR ensemble. Fast Fourier transformation of the ABangle metrics allows to assign timescales of 0.1‐10 GHz to the fastest collective interdomain movements. The results clearly show the necessity of dynamics to understand and characterize the favorable orientations of the V_H_ and V_L_ domains implying a considerable binding interface flexibility and reveal in all antibody fragments (Fab, scFv, and Fv) very similar V_H_‐V_L_ interdomain variations comparable to the distributions observed for known X‐ray structures of antibodies.

**Significance Statement:**

Antibodies have become key players as therapeutic agents. The binding ability of antibodies is determined by the antigen‐binding fragment (Fab), in particular the variable fragment region (Fv). Antigen‐binding is mediated by the complementarity‐determining regions consisting of six loops, each three of the heavy and light chain variable domain V_H_ and V_L_. The relative orientation of the V_H_ and V_L_ domains influences the shape of the antigen‐binding site and is a major objective in antibody design. In agreement with NMR experiments and molecular dynamics simulations, we show a considerable binding site flexibility in the low nanosecond timescale. Thus we suggest that this flexibility and its implications for binding and specificity should be considered when designing and optimizing therapeutic antibodies.

## 1 INTRODUCTION

Antibodies have become an important tool in therapeutics and clinical diagnostics.^1^, ^2^ This increasing relevance has motivated the development of computational techniques to study antibody structure and function.^3^, ^4^ The ability of antibodies to specifically recognize a broad variety of pathogenic molecules is determined by the antigen‐binding fragment (Fab), in particular the variable fragment region (Fv). The Fab consists of a heavy and a light chain that can both be subdivided into a variable (Fv) and a constant region. Fab systems are relatively large and remain a challenge in molecular dynamics simulations. Therefore, various studies only consider the Fv fragment to describe and investigate antigen‐binding. This reduces the system size and thereby decreases the computational time and costs.^5^ The Fv fragment is the focal point of recombination and hypermutation events.^6^, ^7^, ^8^, ^9^, ^10^, ^11^ Antigen‐binding is mediated by six loops of variable sequence and length denoted as the complementarity‐determining regions (CDRs) which are distributed evenly over the heavy and light chain variable domains, V_H_ and V_L_. Besides lengths and sequence of the CDRs, the relative orientation of V_H_ and V_L_ is a third very important factor that determines the shape of the antigen‐binding site.^12^ The variability in orientation of the V_H_ and V_L_ domains to one another is an additional structural feature of antibodies, which directly increases the repertoire of antibody specificity.^13^ Modifications of the V_H_‐V_L_ domain orientation directly change the binding site geometry and have an effect on the specificity of the paratope, the antigen‐binding site, for target antigens.^14^ It has been shown that reducing the system to the variable regions might not always be sufficient to characterize the antigen‐binding process with molecular dynamics simulations, because of possible stabilization in the Fab by C_H_1‐C_L_.^5^ Still, the characterization of the V_H_‐V_L_ domain orientation is crucial in understanding the antigen‐binding process. Antibody‐antigen binding can be understood in terms of the conformational selection mechanism.^15^, ^16^ This paradigm follows the idea of an ensemble of preexisting conformations with different probabilities from which the binding competent state is selected. Transitions between different states in this preexisting conformational space can occur on different timescales, and therefore characterization of the thermodynamics and kinetics is vital to understand their conformational diversity.^17^

**Figure 1:**
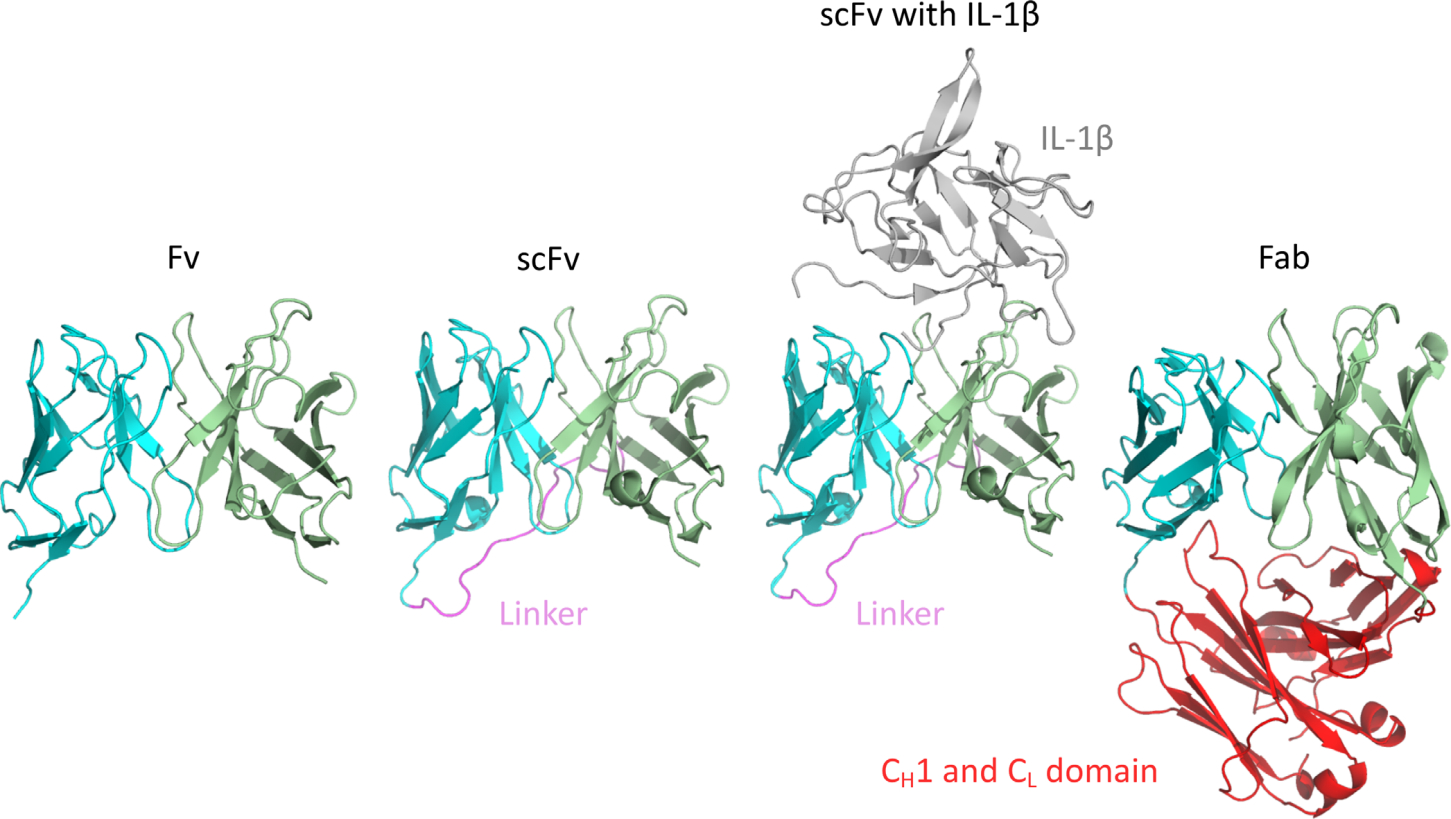
Antibody fragments used as starting structures for the molecular dynamics simulations and the NOE distance restraints simulations. IL, interleukin; scFv, single‐chain variable fragment antibody [Color figure can be viewed at wileyonlinelibrary.com]

This work uses experimental Nuclear Magnetic Resonance (NMR) Nuclear Overhauser Effect (NOE) data in combination with molecular dynamics simulations to understand the V_H_‐V_L_ domain movements. We compare the V_H_‐V_L_ domain orientation observed in our simulations to the NMR ensemble of a single‐chain variable fragment^18^ (scFv) and the corresponding antigen‐binding fragment (Fab). The scFv is the smallest fragment to retain full binding activity and can bind a target protein the same way a Fab does.^19^ The systems studied by simulations with and without NOE time‐averaged restraints are shown in Figure 1. We analyzed this potential therapeutic antibody targeted at the cytokine IL‐1β.^20^ Human IL‐1β is an active pro‐inflammatory cytokine and is a key orchestrator in autoinflammatory and immune responses.^21^ IL‐1β signaling requires the assembly of a heterotrimeric complex consisting of the IL‐1β, the interleukin‐1 receptor type I (IL‐1RI), and the interleukin‐1 receptor accessory protein (IL‐1RAcP). Neutralization of IL‐1β can be achieved with a therapeutic antibody by interfering either with the binding to the IL‐1RI or the interaction between IL‐1β and IL‐1RAcP.^22^

## 2 METHODS

The NOEs and suggested structures for the scFv complex and the Fab were provided by the group of Mark Carr at University Leicester.^19^, ^23^ The first structure of the NMR ensemble of the scFv‐IL‐1β complex with the Protein Data Bank (PDB) Code (2KH2) was used as a starting structure for further simulations. The published NMR ensemble of the complex scFv will be referred to as “minimized NMR ensemble”. In contrast to this, simulations performed with NOE restraints will be referred to as “simulated NMR ensemble.” In addition, we removed IL‐1β and the glycine‐serine linker (G4S) and simulated the scFv and the Fv to understand the influence of complexation and the linker on the flexibility in these angles.

All structures were prepared in MOE (Molecular Operating Environment, Montreal, QC, Canada: 2018)^23^ using the Protonate 3D^24^ tool. The C‐termini of the Fv structures were capped with N‐methylamine. With the tleap tool of the AmberTools16^25^ package, the two systems were placed into cubic water boxes of TIP3P^26^ water molecules with a minimum wall distance to the protein of 10 Å. Parameters for all antibody simulations were derived from the AMBER force field 14SB.^27^ To neutralize the charges, we used uniform background charges. Each system was carefully equilibrated using a multistep equilibration protocol.^28^

### 2.1 Molecular dynamics simulations

The scFv with and without IL‐1β, Fv, and the Fab structures were simulated for 1 μs using molecular dynamics as implemented in the AMBER 18 simulation package.^29^ Molecular dynamics simulations were performed in an NpT ensemble using pmemd.cuda.^30^ Bonds involving hydrogen atoms were restrained by applying the SHAKE algorithm,^31^ allowing a time step of 2.0 fs. Atmospheric pressure of the system was preserved by weak coupling to an external bath using the Berendsen algorithm.^32^ The Langevin thermostat was used to maintain the temperature at 300K during simulations.^33^

### 2.2 NOE restraints simulations—NMR ensemble

The NOE distances are intramolecular NOEs and define the distances between amide protons.^34^ The NOE values were converted on the basis of peak intensities into distances with upper limits of 5.0 Å (strong), 6.5 Å (medium), and 8.0 Å (weak). The structures were minimized, equilibrated, and then simulated for 1 μs using the NOE distance restraints (1141 for the complex scFv and 556 for the Fab) including time‐averaged constraints^34^, ^35^ in an NpT ensemble using pmemd.cuda,^30^ following the same parameters as described in Section 2.1. A time constant for the memory function for the distance restraints of 100 ns was chosen.

### 2.3 ABangle

ABangle^36^ is a computational tool to characterize the relative orientations between the antibody variable domains (V_H_ and V_L_) using six measurements (five angles in degree and one distance in Å of the two domains, Figure S10). A plane is projected on each of the two variable domains. To define these planes, the first two components of a principal component analysis of 240 reference coordinates were used for V_H_ and V_L_ each. The reference coordinate set consists of Cα coordinates of eight conserved residues (as identified by Abhinandan^12^) for 30 cluster representatives from a sequence clustering of the nonredundant ABangle antibody data set. The planes were then fit through those 240 coordinates, and consensus structures consisting of 35 structurally conserved Cα positions were created for the V_H_ and V_L_ domain.^36^ Between those two planes, a distance vector C is defined. The six measures are then two tilt angles between each plane (HC1, HC2, LC1, LC2) and a torsion angle (HL) between the two planes along the distance vector (dc). These angles are visualized on the following link of the Oxford Protein Informatics Group website (http://www.stats.ox.ac.uk/~dunbar/abangle/wobble.html). The ABangle script can calculate these measures for an arbitrary Fv region by aligning the consensus structures to the found core set positions and fitting the planes and distance vector from this alignment. This tool available online was combined with an in‐house python script to reduce computational time and to visualize our simulation data over time. The in‐house script makes use of ANARCI^37^ for fast local annotation of the Fv region and pytraj^38^ for rapid trajectory processing. The resulting time‐dependent ABangle^36^ plots were color‐coded according to the size of the antibody fragment used in the simulation and the inclusion of experimental data. In the background, relative domain orientations observed in a representative data set of the PDB are displayed. The resulting fluctuations in these six measures were further analyzed with a Fast Fourier Transformation (FFT)^39^ in python^40^, ^41^ to characterize the frequency and timescale of these movements. We applied a frequency filter to assign timescales to movements.

## 3 RESULTS

**Figure 2:**
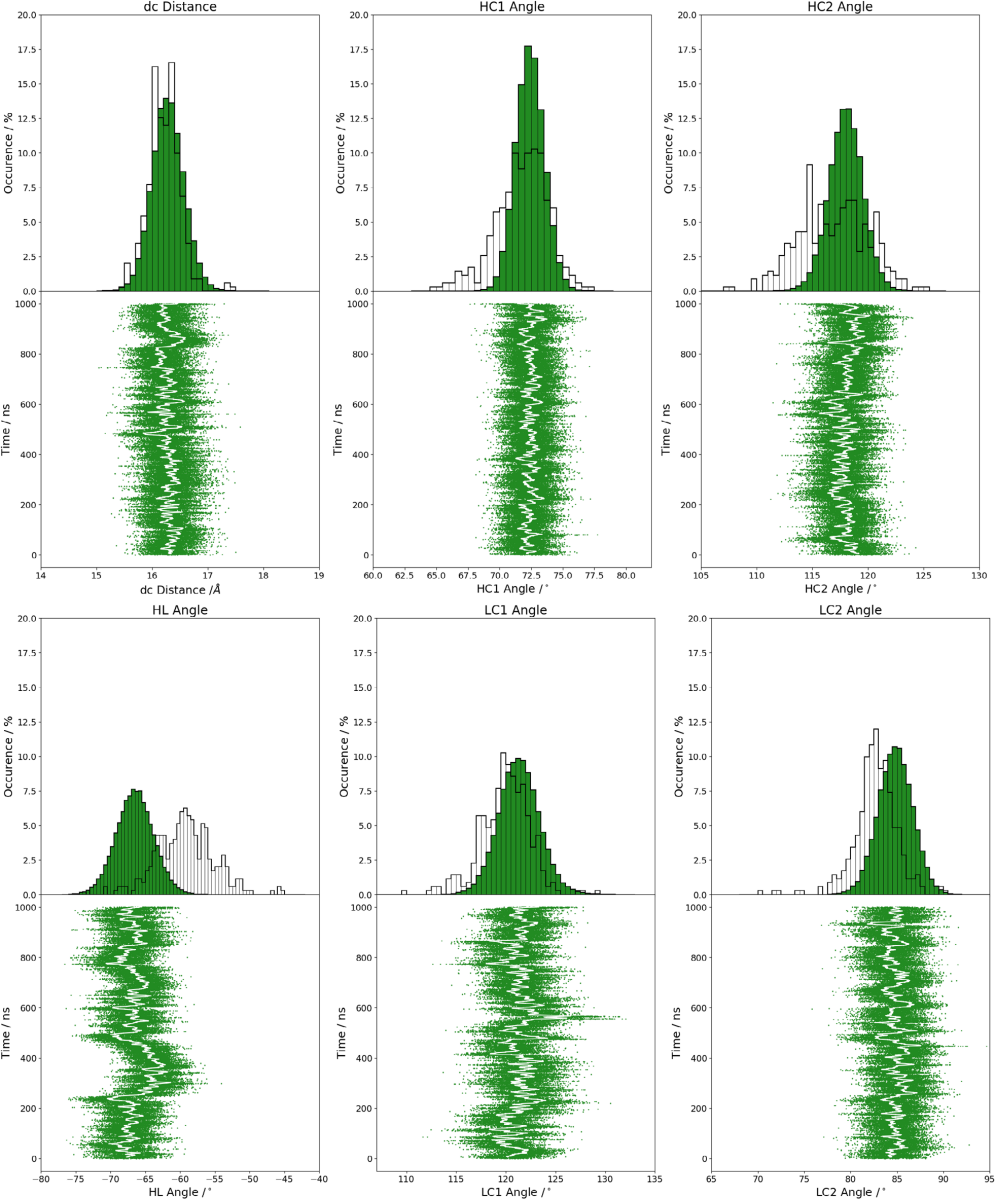
ScFv without the presence of IL‐1β. ABangle measures of 1‐μs molecular dynamics simulation with the published ABangle PDB distribution in the background. IL, interleukin; scFv, single‐chain variable fragment antibody [Color figure can be viewed at wileyonlinelibrary.com]

**Figure 3:**
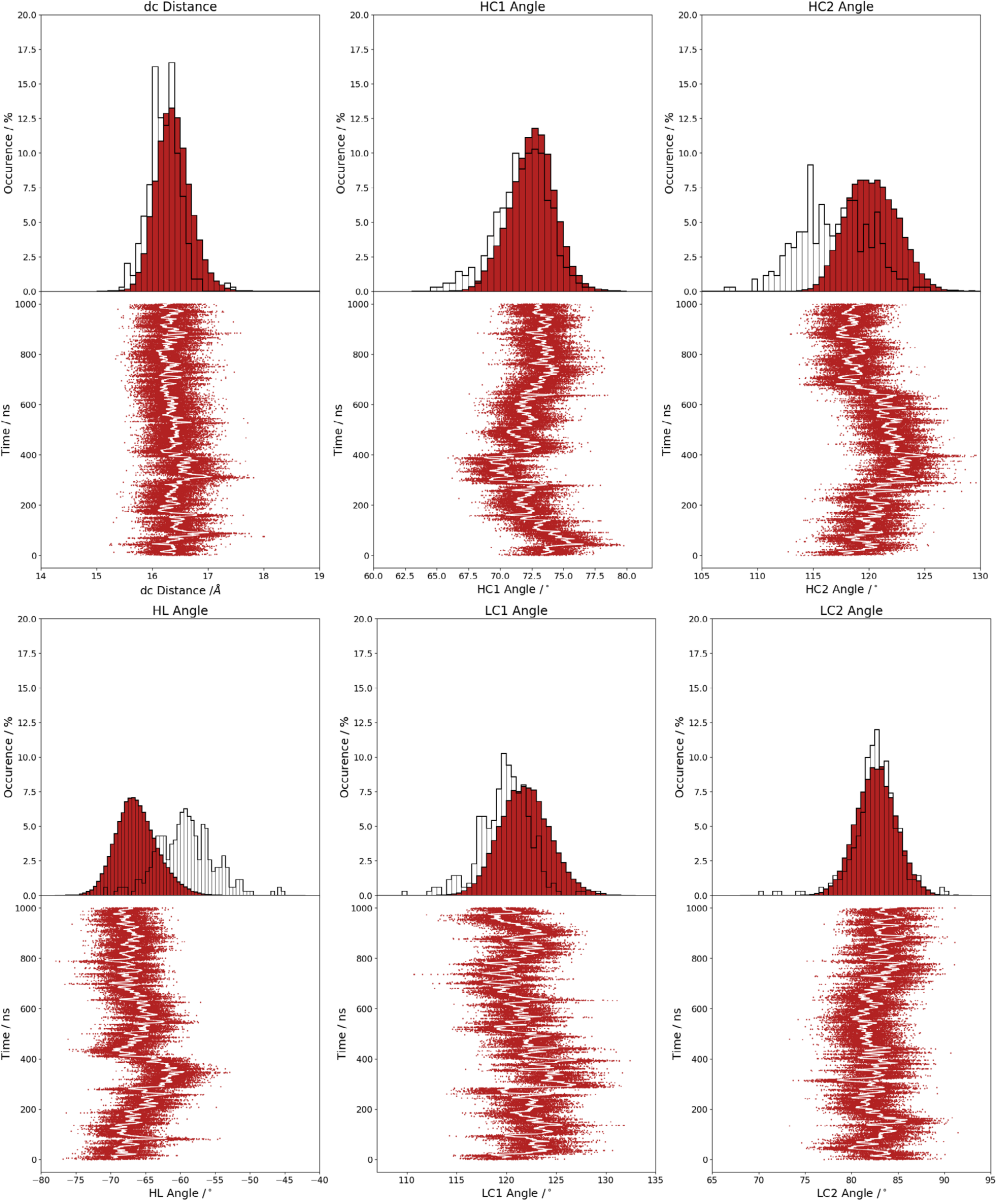
Complex scFv ABangle measure of 1‐μs NOE time‐averaged restraints molecular dynamics simulation (NMR constraints are taken from the complex, the simulation was performed in complex with the antigen). scFv, single‐chain variable fragment antibody [Color figure can be viewed at wileyonlinelibrary.com]

**Figure 4:**
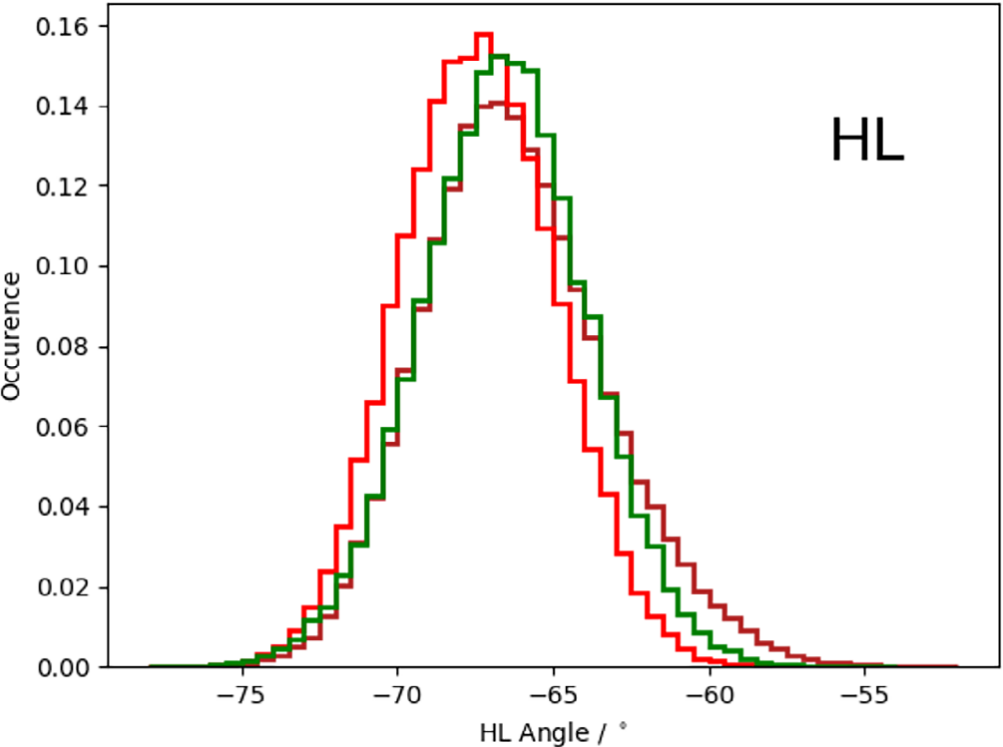
HL angle distribution overlays for the complex scFv simulated with and without time‐averaged NOE distance restraints and molecular dynamics simulation of the scFv without the presence of antigen. scFv, single‐chain variable fragment antibody [Color figure can be viewed at wileyonlinelibrary.com]

**Figure 5:**
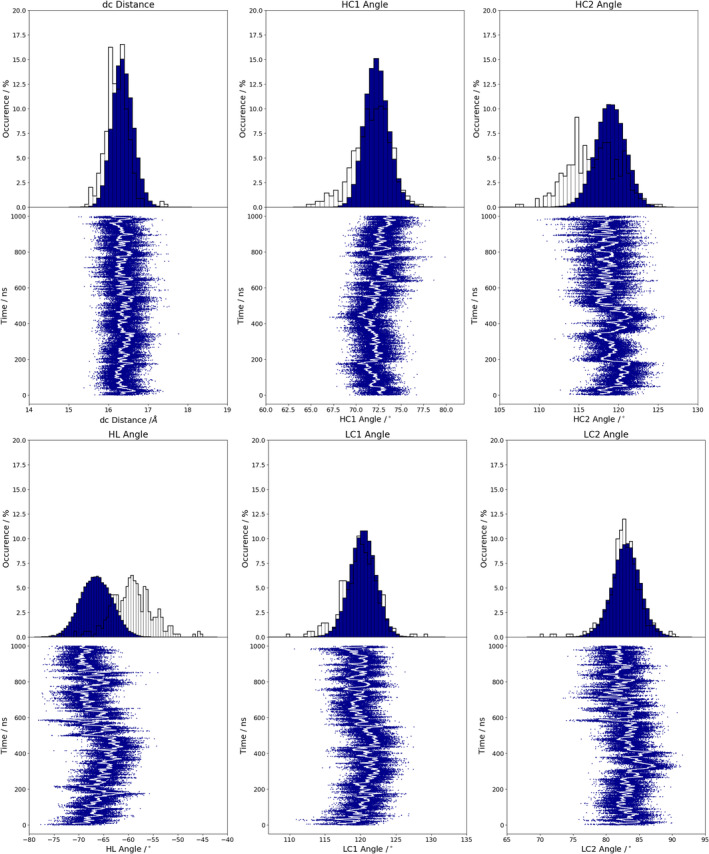
Fab ABangle measures of 1‐μs NOE time‐averaged restraints molecular dynamics simulation. Fab, antigen‐binding fragment [Color figure can be viewed at wileyonlinelibrary.com]

**Figure 6:**
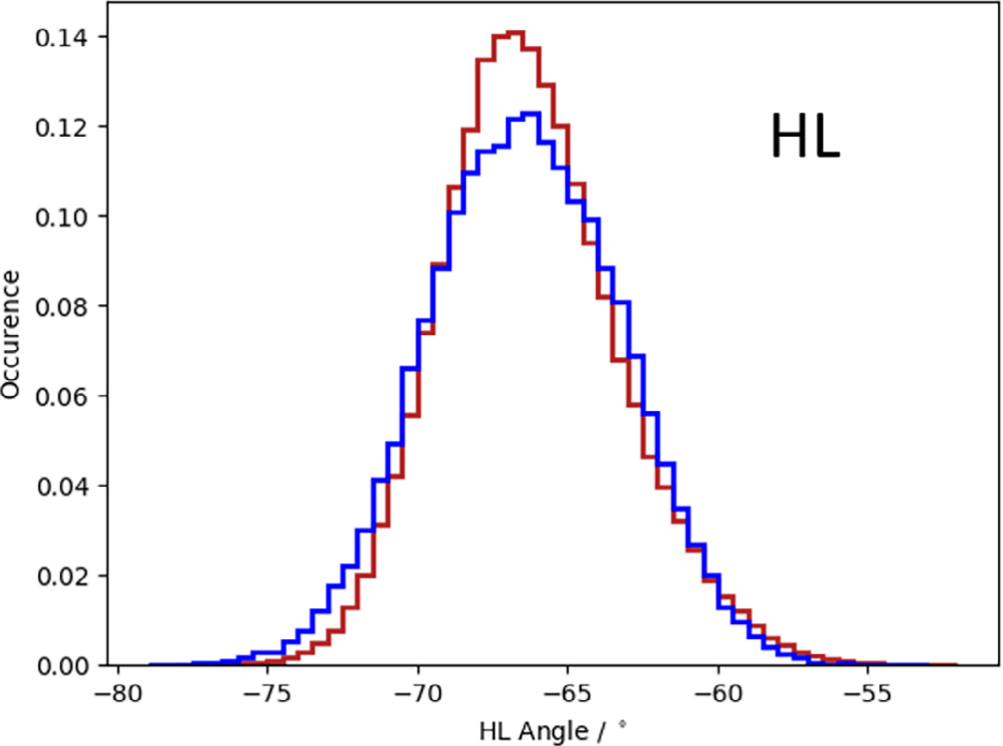
Overlay of the HL angle distributions of the complex (dark red) and the Fab (blue) simulated with NOE time‐averaged restraints. Fab, antigen‐binding fragment [Color figure can be viewed at wileyonlinelibrary.com]

**Figure 7:**
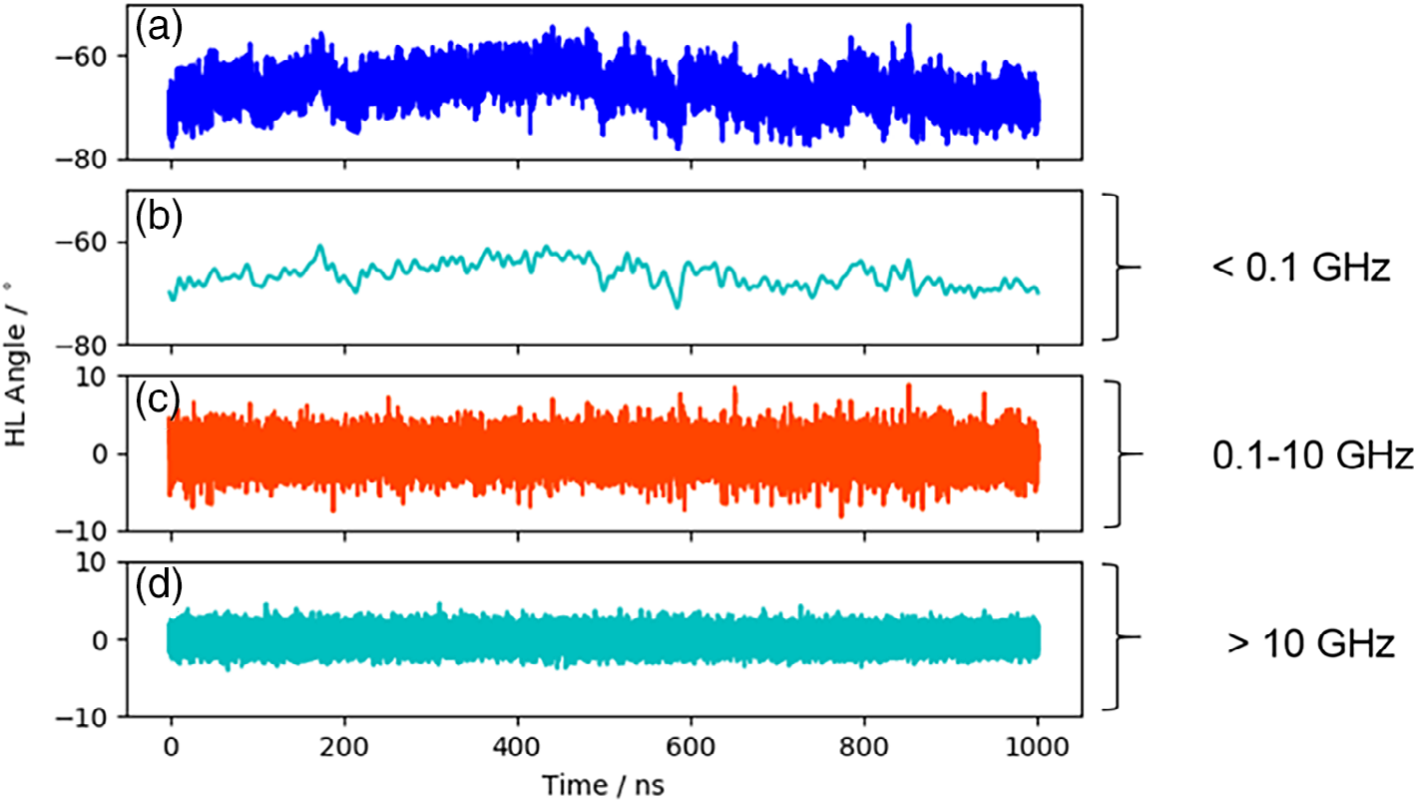
Calculated variances displaying different frequency ranges. A, ABangle HL angle fluctuations of 1‐μs NMR time‐averaged restraints simulation. B, Angle variations of the domain movements occurring in the timescale slower than 10 ns. C, Angle variations capturing the domain movements occurring in the 0.1‐ns and 10‐ns timescale. D, Angle domain variations occurring faster than 0.1 ns [Color figure can be viewed at wileyonlinelibrary.com]

**Figure 8:**
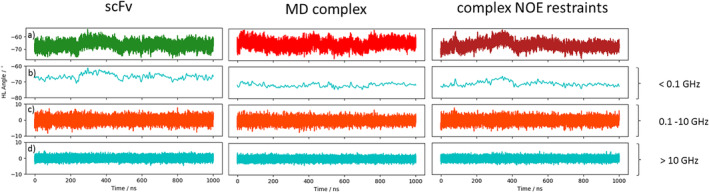
Calculated HL angle variances displaying different frequency ranges. A, ABangle HL angle distributions for the scFv MD simulation, complex MD simulation, and the complex NOE restraints simulation. B, Angle variations of the domain movements occurring in the timescale slower than 10 ns. C, Angle variations capturing the domain movements occurring in the 0.1‐ns and 10‐ns timescale. D, Angle domain variations occurring faster than 0.1 ns. MD, molecular dynamics; scFv, single‐chain variable fragment antibody [Color figure can be viewed at wileyonlinelibrary.com]

Figure S1 illustrates the analysis of the minimized NMR ensemble of the scFv with ABangle and reveals high variations of the V_H_‐V_L_ domain orientation in these six metrics. These high variations are obvious in comparison to the distributions of angles and the distance of the originally published 352 crystal structures found in the PDB displayed in the background. Comparison of 1‐μs molecular dynamics simulations of the scFv without the presence of the IL‐1β (Figure 2) with the observed relative domain orientations in the PDB reveals time fluctuations of these six measures in a similar range as observed for the 352 crystal structures. The fluctuations indicate that majority of the V_H_‐V_L_ movements occur on the nanosecond timescale. This timescale is orders of magnitude faster than that of the loop dynamics, especially of the CDR‐H3 loop, which occur on the microsecond to millisecond timescales.^11^ To characterize the role of the peptide linker on the V_H_‐V_L_ domain orientation, we show the analysis of 1‐μs molecular dynamics simulations without the presence of the linker in Figure S2. We observe in this example that the calculated distributions do not change in the absence of the linker. To analyze the influence of the antigen on the relative V_H_‐V_L_ orientation, the complex scFv was simulated with molecular dynamics simulations and the results are illustrated in Figure S3. To obtain a simulated NMR ensemble, the provided NOEs of the scFv complex were used and the results are shown in Figure 3. Figure 3 shows in all metrics a very similar distribution as observed without linker, without NOE distance restraints, without the presence of antigen and confirms the high variability in the relative domain orientations. The overlay of the HL distribution of the scFv with the Fv is illustrated in Figure S7 and reveals a very similar distribution. To underline very similar distributions, Figure 4 compares the simulations with and without the presence of IL‐1β and with and without NOE distance restraints. It clearly shows that the relative interdomain dynamics captured without antigen follow the conformational selection paradigm, because we seem to find the dynamics involved in antigen binding. Figure S6 compares the experimental NOE upper limits with the calculated NOEs of the scFv NOE restraint simulation and the molecular dynamics simulations with and without antigen bound. The results show with and without restraints and with and without the presence of antigens similar NOEs compared to the experiments. The Fab was analyzed to identify the influence of the C_H_1 and C_L_ domains on the relative domain orientations and compared it with the other analyzed fragments. One‐microsecond molecular dynamics simulations of the Fab model were performed, and the resulting relative interdomain movement distributions are shown in Figure S4. Figure 5 illustrates the time‐averaged NOE restraints simulation of the Fab. The resulting simulated NMR Fab ensemble strengthens the assumption that the ABangle measures describe the same interdomain movements in all considered antibody fragments. Figure 6 directly shows the comparison of the HL angle distributions of the NOE time‐averaged restraints simulations of the complex scFv and the Fab. The same domain orientations can be observed in this example with and without the presence of the constant domains. The excellent statistical sampling of these domain movements allows us to characterize frequencies and timescales of these dynamics by applying FFT. Figure 7, Figure S5, and Figure S9 show the fast Fourier transformed HL angle distributions of the NOE time‐averaged Fab simulations. The spectrum in Figure S5 shows significant peaks in the frequency range of 0.1 to 10 GHz, colored in orange. The spectrum was filtered for the frequency range and back‐transformed to see which domain movements occur (Figure 7C) between 0.1 and 10 ns. The histogram in Figure S10 describes an overlay of the ABangle HL distribution and the filtered movements of the FFT spectrum. The relative domain dynamics captured in 0.1 to 10 ns represent the main interdomain movements and describe about 90% of the observed variance. The remaining 10% can be characterized by movements occurring faster than 0.1 ns or slower than 10 ns. The movements faster than 0.1 ns are shown in Figure 7D, while the dynamics slower than 10 ns are displayed in Figure 7B. Figure 8 compares timescales of movements and amplitudes of motions for the scFv, the complex, and the simulated NMR ensemble for the complex. However, no significant differences neither in the amplitude nor in the movements could be identified.

## 4 DISCUSSION

In the present work, we characterize the relative V_H_‐V_L_ domain orientations in Fab, Fv, and scFv of an antibody with and without the presence of the antigen, IL‐1β, with molecular dynamics simulations and observe in agreement with NMR results the same dynamics in orientations independent of the presence of the linker and the constant region.

Previous studies focused on understanding and quantifying the interdomain V_H_‐V_L_ orientations in antibodies.^12^, ^13^, ^36^, ^42^, ^43^ Predictions of the relative V_H_‐V_L_ domain orientation in antibody design are challenging due to the variations observed.^42^, ^43^ Pauling and Landsteiner in the 1930s and Milstein and Foote in 1994 suggested the ability of the same antibody to adopt various conformations on their binding properties and on the increasing size of the antibody repertoire.^17^, ^44^ Understanding the role of the C_H_1 and C_L_ domains is crucial in characterizing the antigen‐binding process and has therefore been targeted by various experimental and computational studies.^45^ The presence of the constant domains C_H_1 and C_L_ in the Fab has been discussed to have stability benefits in terms of interdomain orientation;^5^ however, this also might be a consequence of too short timescales considered. NMR experiments directly compared the complexed scFv with a Fab and provide evidence that scFvs bind target proteins identically to Fabs.^19^ Combining experimental NOE data with molecular dynamics simulations reveals in this example the same fast interdomain dynamics in the low nanosecond timescale for the different fragments analyzed. The timescale of interdomain reorientation is orders of magnitudes faster than the loop dynamics reflecting a hydrophobic interface with rather unspecific interactions that can be easily broken and result in low kinetic barriers, whereas loop dynamics is dominated by reorganization of hydrogen bond networks and electrostatic interactions characterized by strong forces and high barriers leading to much longer transition time scales.^11^, ^46^ The distributions of interdomain orientations resulting from the time evolutions are very similar to distributions observed for antibody fragments in the PDB.

Table S1 shows the time averages and standard deviations of all the considered antibody fragments and supports the observations that in all simulations we characterize a similar domain movement. Figures 3 and 5 present the results for 1‐μs NOE time‐averaged restraints molecular dynamics simulations and display very similar angle and distance distributions over the 1‐μs simulation time (Figure 6). Comparison of the Fv with the scFv distributions in Figure S2 and Figure 2 describes independent of the presence of the linker the same domain dynamics and show very similar angle and distance variances (Table S1 and Figure S6). The main differences between all antibody fragments with and without NOE time‐averaged restraints is in the HC2 angle, where the introduced NOEs lead to a more right‐shifted distribution. However, HC2 angles observed in the molecular dynamics simulations overlap considerably with the simulated NMR distributions (Figure S8). The shape of the overall angle fluctuations (Figure 7, Figure S5 and Figure S9) is dominated by dynamics occurring on frequencies <0.1 GHz. Thus this could be the functionally relevant dynamics, whereas faster motions are harmonic fluctuations that can or cannot be coupled to slower dynamics. Figure S9 clearly points out that in terms of frequencies of motions, the HL angle distribution is again dominated by movements slower than 10 ns, although no substantial differences in amplitude with and without experimental restraints and upon binding could be identified.

## 5 CONCLUSION

In this study, the combination of experimental NOE data with molecular dynamics simulations reveals fast interdomain dynamics with very similar angle and distance distributions independent of the presence of the linker, C_H_1‐C_L_ domain, and the binding partner IL‐1β. The relative domain orientations can vary about ±5 degrees in the angles and about ± 1 Å in the distance in less than 1 ns. These results are in agreement with the experimental NMR data and confirm the high interdomain dynamics of Fvs, scFvs, and Fabs. These findings are important for antibody structure prediction, as short molecular dynamics simulations are already sufficient to capture the majority of possible interdomain orientations. The fast conformational transitions of these relative domain orientations occur in the low nanosecond timescale. Therefore, these measurements should not be considered as static metrics. Current tools for predicting the VH‐VL domain orientation provide an important starting point for antibody design. However, already short simulations allow accessing an ensemble of relevant domain orientations potentially involved in antigen binding.

## AUTHOR CONTRIBUTIONS

The manuscript was discussed and written through contributions of all authors. All authors have given approval to the final version of the manuscript.

## CONFLICT OF INTEREST

The authors declare no potential conflict of interest.

## Supporting information


**Figure S1** ABangle results of the minimized complex scFv NMR ensemble combined with the PDB distribution colored in gray.
**Figure S2:** Fv ABangle measure of 1 μs molecular dynamics simulation (in the absence of the linker).
**Figure S3:** Complex scFv ABangle measure of 1 μs molecular dynamics simulation.
**Figure S4:** Fab ABangle measures of 1 μs molecular dynamics simulation.
**Figure S5:** FFT Spectrum of the HL angle fluctuations of the Fab simulated NMR ensemble.
**Figure S6:** Comparison of experimental scFv complex NOEs with the calculated NOEs of 1 μs simulations with and without the antigen bound and with the complex NOE restraints. The plot shows the scFv fragment. The gap is caused by the peptide (G4S) linker, which directly connects the light and the heavy chain variable domains.
**Figure S7:** Overlay of the HL angle distributions of the Fv (green) and the scFv (forestgreen) fragments.
**Figure S8:** Overlay of the HC2 angle distributions of the Fv (green) and the NOE Fab simulations (blue) fragments
**Table S1:** Average and standard deviations of the six ABangle measures for all six considered antibody fragments.
**Figure S9:** Overlay of the ABangle histogram (blue) with the angle variations observed in the 0.1 to 10 ns timescale (orange).
**Figure S10:** Illustration of the ABangle angle and distance definitions.

## References

[1] Reichert JM . Antibodies to watch in 2017. MAbs. 2016;9:167‐181. 27960628 10.1080/19420862.2016.1269580 PMC5297518

[2] Pavlou AK , Belsey MJ . The therapeutic antibodies market to 2008. Eur J Pharm Biopharm. 2005;59:389‐396. 15760719 10.1016/j.ejpb.2004.11.007

[3] Kuroda D , Shirai H , Jacobson MP , Nakamura H . Computer‐aided antibody design. Protein Eng Des Sel. 2012;25:507‐522. 22661385 10.1093/protein/gzs024 PMC3449398

[4] Clark LA , Ganesan S , Papp S , van Vlijmen HWT . Trends in antibody sequence changes during the somatic hypermutation process. J Immunol. 2006;177:333‐340. 16785529 10.4049/jimmunol.177.1.333

[5] Knapp B , Dunbar J , Alcala M , Deane CM . Variable regions of antibodies and T‐cell receptors may not be sufficient in molecular simulations investigating binding. J Chem Theory Comput. 2017;13:3097‐3105. 28617587 10.1021/acs.jctc.7b00080

[6] Teng G , Papavasiliou FN . Immunoglobulin somatic hypermutation. Annu Rev Genet. 2007;41:107‐120. 17576170 10.1146/annurev.genet.41.110306.130340

[7] Chothia C , Lesk AM . Canonical structures for the hypervariable regions of immunoglobulins. J Mol Biol. 1987;196:901‐917. 3681981 10.1016/0022-2836(87)90412-8

[8] Fennell BJ , McDonnell B , Tam ASP , et al. CDR‐restricted engineering of native human scFvs creates highly stable and soluble bifunctional antibodies for subcutaneous delivery. MAbs. 2013;5:882‐895. 23995618 10.4161/mabs.26201 PMC3896602

[9] Dimitrov JD , Pashov AD , Vassilev TL . Antibody polyspecificity Naturally Occurring Antibodies (NAbs). Paris, France: Springer; 2012:213‐226. 10.1007/978-1-4614-3461-0_16 22903677

[10] Dimitrov JD , Lacroix‐Desmazes S , Kaveri SV , Vassilev TL . Transition towards antigen‐binding promiscuity of a monospecific antibody. Mol Immunol. 2007;44:1854‐1863. 17097144 10.1016/j.molimm.2006.10.002

[11] Fernández‐Quintero ML , Loeffler JR , Kraml J , Kahler U , Kamenik AS , Liedl KR . Characterizing the diversity of the CDR‐H3 loop conformational ensembles in relationship to antibody binding properties. Front Immunol. 2019;9:3065. 30666252 10.3389/fimmu.2018.03065 PMC6330313

[12] Abhinandan KR , Martin ACR . Analysis and prediction of VH/VL packing in antibodies. Protein Eng des Sel. 2010;23:689‐697. 20591902 10.1093/protein/gzq043

[13] Bujotzek A , Dunbar J , Lipsmeier F , et al. Prediction of VH–VL domain orientation for antibody variable domain modeling. Proteins. 2015;83:681‐695. 25641019 10.1002/prot.24756

[14] Bujotzek A , Lipsmeier F , Harris SF , Benz J , Kuglstatter A , Georges G . VH‐VL orientation prediction for antibody humanization candidate selection: a case study. MAbs. 2016;8:288‐305. 26637054 10.1080/19420862.2015.1117720 PMC4966660

[15] Csermely P , Palotai R , Nussinov R . Induced fit, conformational selection and independent dynamic segments: an extended view of binding events. Trends Biochem Sci. 2010;35:539‐546. 20541943 10.1016/j.tibs.2010.04.009 PMC3018770

[16] Tsai C‐J , Kumar S , Ma B , Nussinov R . Folding funnels, binding funnels, and protein function. Protein Sci. 1999;8:1181‐1190. 10386868 10.1110/ps.8.6.1181 PMC2144348

[17] Foote J , Milstein C . Conformational isomerism and the diversity of antibodies. Proc Natl Acad Sci U S A. 1994;91:10370‐10374. 7937957 10.1073/pnas.91.22.10370 PMC45021

[18] Bird R , Hardman K , Jacobson J , et al. Single‐chain antigen‐binding proteins. Science. 1988;242:423‐426. 3140379 10.1126/science.3140379

[19] Wilkinson IC , Hall CJ , Veverka V , et al. High resolution NMR‐based model for the structure of a scFv‐IL‐1β complex: potential for NMR as a key tool in therapeutic antibody design and development. J Biol Chem. 2009;284:31928‐31935. 19776018 10.1074/jbc.M109.025304 PMC2797264

[20] Lopez‐Castejon G , Brough D . Understanding the mechanism of IL‐1β secretion. Cytokine Growth Factor Rev. 2011;22:189‐195. 22019906 10.1016/j.cytogfr.2011.10.001 PMC3714593

[21] Lee CV , Koenig P , Fuh G . A two‐in‐one antibody engineered from a humanized interleukin 4 antibody through mutation in heavy chain complementarity‐determining regions. MAbs. 2014;6:622‐627. 24618680 10.4161/mabs.28483 PMC4011906

[22] Church LD , McDermott MF . Canakinumab: a human anti‐IL‐1β monoclonal antibody for the treatment of cryopyrin‐associated periodic syndromes. Expert Rev Clin Immunol. 2010;6:831‐841. 20979548 10.1586/eci.10.66

[23] Addis PW , Hall CJ , Bruton S , et al. Conformational heterogeneity in antibody‐protein antigen recognition: implications for high affinity protein complex formation. J Biol Chem. 2014;289:7200‐7210. 24436329 10.1074/jbc.M113.492215 PMC3945379

[24] Labute P . Protonate3D: assignment of ionization states and hydrogen coordinates to macromolecular structures. Proteins. 2009;75:187‐205. 18814299 10.1002/prot.22234 PMC3056144

[25] D.A. Case , R.M. Betz , D.S. Cerutti , T.E. Cheatham, III , T.A. Darden , R.E. Duke , T.J. Giese , H. Gohlke , A.W. Goetz , N. Homeyer , S. Izadi , P. Janowski , J. Kaus , A. Kovalenko , T.S. Lee , S. LeGrand , P. Li , C., Lin, T. Luchko, R. Luo, B. Madej, D. Mermelstein, K.M. Merz, G. Monard, H. Nguyen, H.T. Nguyen, I. , Omelyan, A. Onufriev, D.R. Roe, A. Roitberg, C. Sagui, C.L. Simmerling, W.M. Botello‐Smith, J. Swails, R.C. Walker, J. Wang, R.M. Wolf, X. Wu , L. Xiao and P.A. Kollman (2016), AMBER 2016, University of California, San Francisco, 2016

[26] Jorgensen WL , Chandrasekhar J , Madura JD , Impey RW , Klein ML . Comparison of simple potential functions for simulating liquid water. J Chem Phys. 1983;79:926‐935.

[27] Maier JA , Martinez C , Kasavajhala K , Wickstrom L , Hauser KE , Simmerling C . ff14SB: improving the accuracy of protein side chain and backbone parameters from ff99SB. J Chem Theory Comput. 2015;11:3696‐3713. 26574453 10.1021/acs.jctc.5b00255 PMC4821407

[28] Wallnoefer HG , Liedl KR , Fox T . A challenging system: free energy prediction for factor Xa. J Comput Chem. 2011;32:1743‐1752. 21374633 10.1002/jcc.21758

[29] D.A. Case , R.M. Betz , D.S. Cerutti , T.E. Cheatham, III , T.A. Darden , R.E. Duke , T.J. Giese , H. Gohlke , A.W. Goetz , N. Homeyer , S. Izadi , P. Janowski , J. Kaus , A. Kovalenko , T.S. Lee , S. LeGrand , P. Li , C., Lin, T. Luchko, R. Luo, B. Madej, D. Mermelstein, K.M. Merz, G. Monard, H. Nguyen, H.T. Nguyen, I. , Omelyan, A. Onufriev, D.R. Roe, A. Roitberg, C. Sagui, C.L. Simmerling, W.M. Botello‐Smith, J. Swails, R.C. Walker, J. Wang, R.M. Wolf, X. Wu , L. Xiao and P.A. Kollman (2016), AMBER 2018, University of California, San Francisco, 2016.

[30] Salomon‐Ferrer R , Götz AW , Poole D , Le Grand S , Walker RC . Routine microsecond molecular dynamics simulations with AMBER on GPUs. 2. Explicit solvent particle mesh Ewald. J Chem Theory Comput. 2013;9:3878‐3888. 26592383 10.1021/ct400314y

[31] Miyamoto S , Kollman PA . Settle: an analytical version of the SHAKE and RATTLE algorithm for rigid water models. J Comput Chem. 1992;13:952‐962.

[32] Berendsen H , van Postma JPM , van Gunsteren W , DiNola A , Haak JR . Molecular dynamics with coupling to an external bath. J Chem Phys. 1984;81:3684.

[33] J. D. Doll , E. Myers L , Adelman S. Generalized Langevin equation approach for atom/solid‐surface scattering: Inelastic studies. 1975 J Chem Phys 63 4908.

[34] Palmer AG . NMR characterization of the dynamics of biomacromolecules. Chem Rev. 2004;104:3623‐3640. 15303831 10.1021/cr030413t

[35] Zagrovic B , van Gunsteren WF . Comparing atomistic simulation data with the NMR experiment: how much can NOEs actually tell us? Proteins 2006; 63:210–8. 16425239 10.1002/prot.20872

[36] Dunbar J , Fuchs A , Shi J , Deane CM . ABangle: characterising the VH–VL orientation in antibodies. Protein Eng Des Sel. 2013;26:611‐620. 23708320 10.1093/protein/gzt020

[37] Dunbar J , Deane CM . ANARCI: antigen receptor numbering and receptor classification. Bioinformatics. 2016;32:298‐300. 26424857 10.1093/bioinformatics/btv552 PMC4708101

[38] David A . Case HN , Daniel R . Roe JS. PYTRAJ: interactive data analysis for molecular dynamics simulations. 2016.

[39] Bergland GD . A guided tour of the fast Fourier transform. IEEE Spectrum. 1969;6:41‐52.

[40] Millman KJ , Aivazis M . Python for scientists and engineers. Comput Sci Eng. 2011;13:9‐12.

[41] Oliphant TE . Python for scientific computing. Comput Sci Eng. 2007;9:10‐20.

[42] Weitzner B , R Jeliazkov J , Lyskov S , Marze N , Kuroda D , Frick R , Adolf‐Bryfogle J , Biswas N , Dunbrack R , Gray J. Modeling and docking of antibody structures with Rosetta 2017 Nat Protoc 12:401‐416. 28125104 10.1038/nprot.2016.180 PMC5739521

[43] Marze NA , Lyskov S , Gray JJ . Improved prediction of antibody VL–VH orientation. Protein Eng Des Sel. 2016;29:409‐418. 27276984 10.1093/protein/gzw013 PMC5036862

[44] Pauling L . A theory of the structure and process of formation of antibodies. J Am Chem Soc. 1940;62:2643‐2657.

[45] Röthlisberger D , Honegger A , Plückthun A . Domain interactions in the fab fragment: a comparative evaluation of the single‐chain Fv and fab format engineered with variable domains of different stability. J Mol Biol. 2005;347:773‐789. 15769469 10.1016/j.jmb.2005.01.053

[46] Fernández‐Quintero ML , Kraml J , Georges G , Liedl KR . CDR‐H3 loop ensemble in solution—conformational selection upon antibody binding. MAbs. 2019;11:1077‐1088. 31148507 10.1080/19420862.2019.1618676 PMC6748594

