# Supplementary material for: VH‐VL interdomain dynamics observed by computer simulations and NMR
Source: Proteins. 2020 Jan 14;88(7):830–9. doi: 10.1002/prot.25872 (PMC7317758; doi:10.1002/prot.25872)
Supplement: Supplementary file 1 — Figure S1 ABangle results of the minimized complex scFv NMR ensemble combined with the PDB distribution colored in gray. Figure S2: Fv ABangle measure of 1 μs molecular dynamics simulation (in the absence of the linker). Figure S3: Complex scFv ABangle measure of 1 μs molecular dynamics simulation. Figure S4: Fab ABangle measures of 1 μs molecular dynamics simulation. Figure S5: FFT Spectrum of the HL angle fluctuations of the Fab simulated NMR ensemble. Figure S6: Comparison of experimental scFv complex NOEs with the calculated NOEs of 1 μs simulations with and without the antigen bound and with the complex NOE restraints. The plot shows the scFv fragment. The gap is caused by the peptide (G4S) linker, which directly connects the light and the heavy chain variable domains. Figure S7: Overlay of the HL angle distributions of the Fv (green) and the scFv (forestgreen) fragments. Figure S8: Overlay of the HC2 angle distributions of the Fv (green) and the NOE Fab simulations (blue) fragments Table S1: Average and standard deviations of the six ABangle measures for all six considered antibody fragments. Figure S9: Overlay of the ABangle histogram (blue) with the angle variations observed in the 0.1 to 10 ns timescale (orange). Figure S10: Illustration of the ABangle angle and distance definitions. [file PROT-88-830-s001.docx]

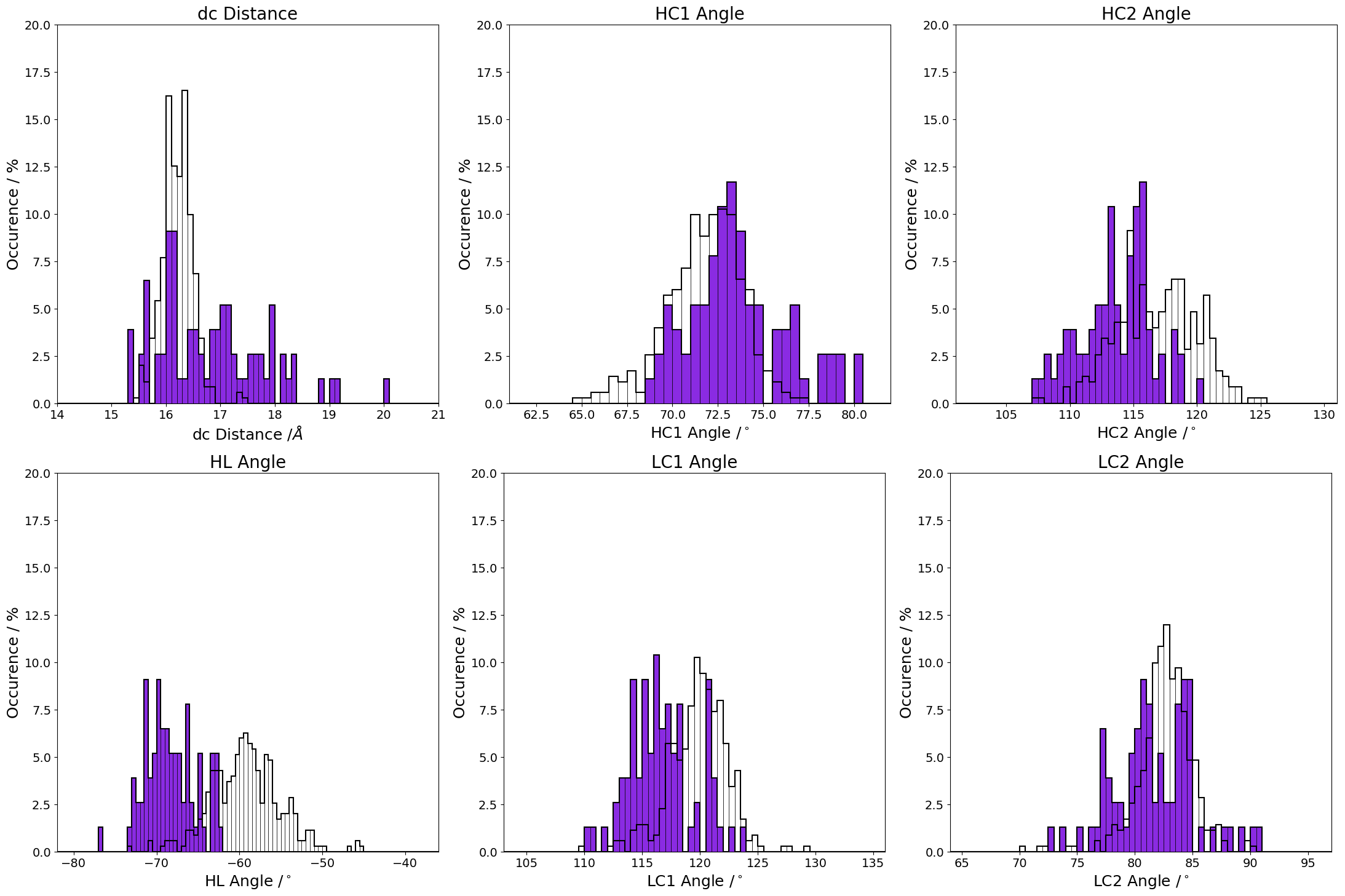


SI Figure S1: ABangle results of the minimized complex scFv NMR ensemble combined with the PDB distribution colored in gray.


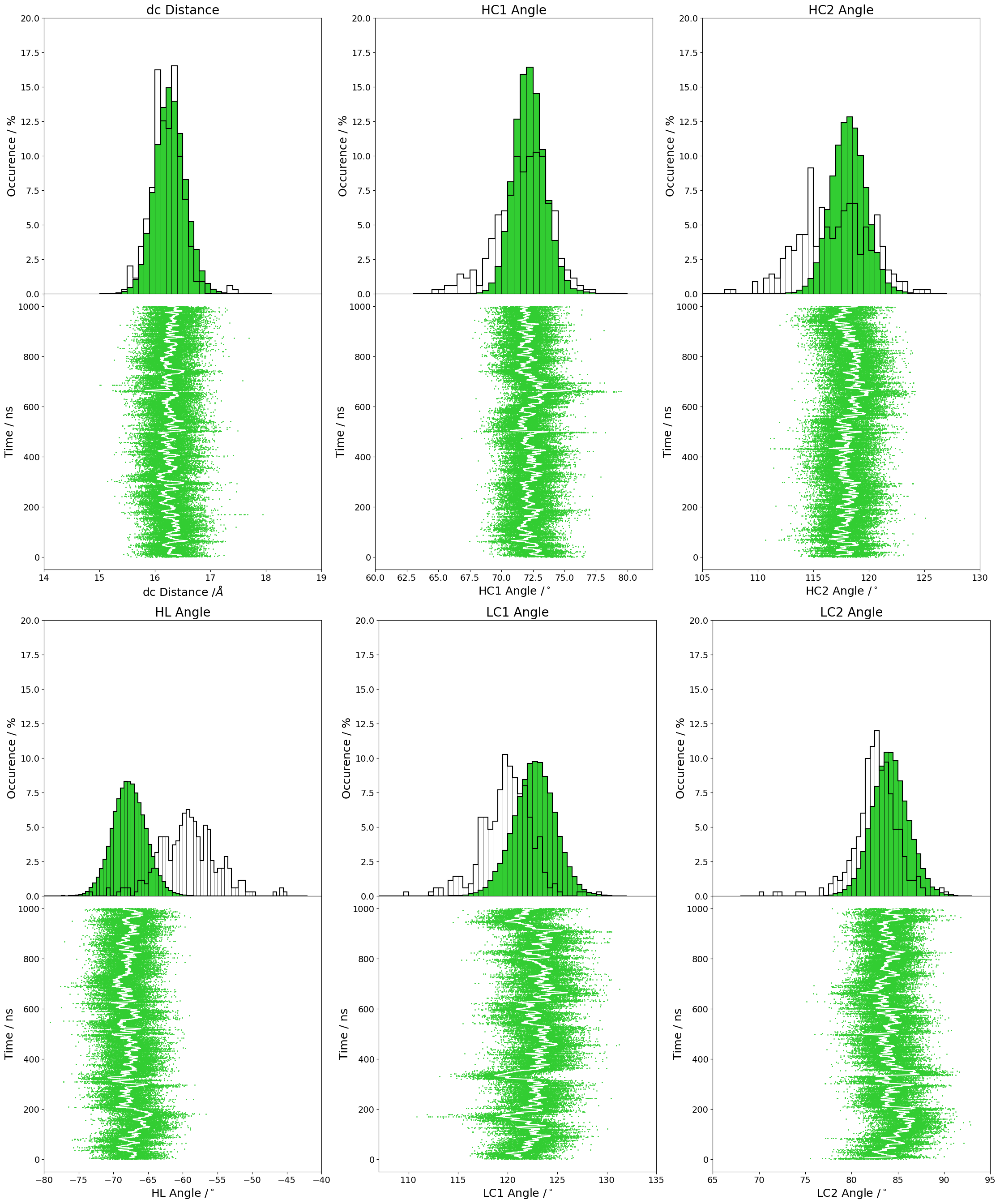
 SI Figure S2: Fv ABangle measure of 1 µs molecular dynamics simulation (in the absence of the linker).


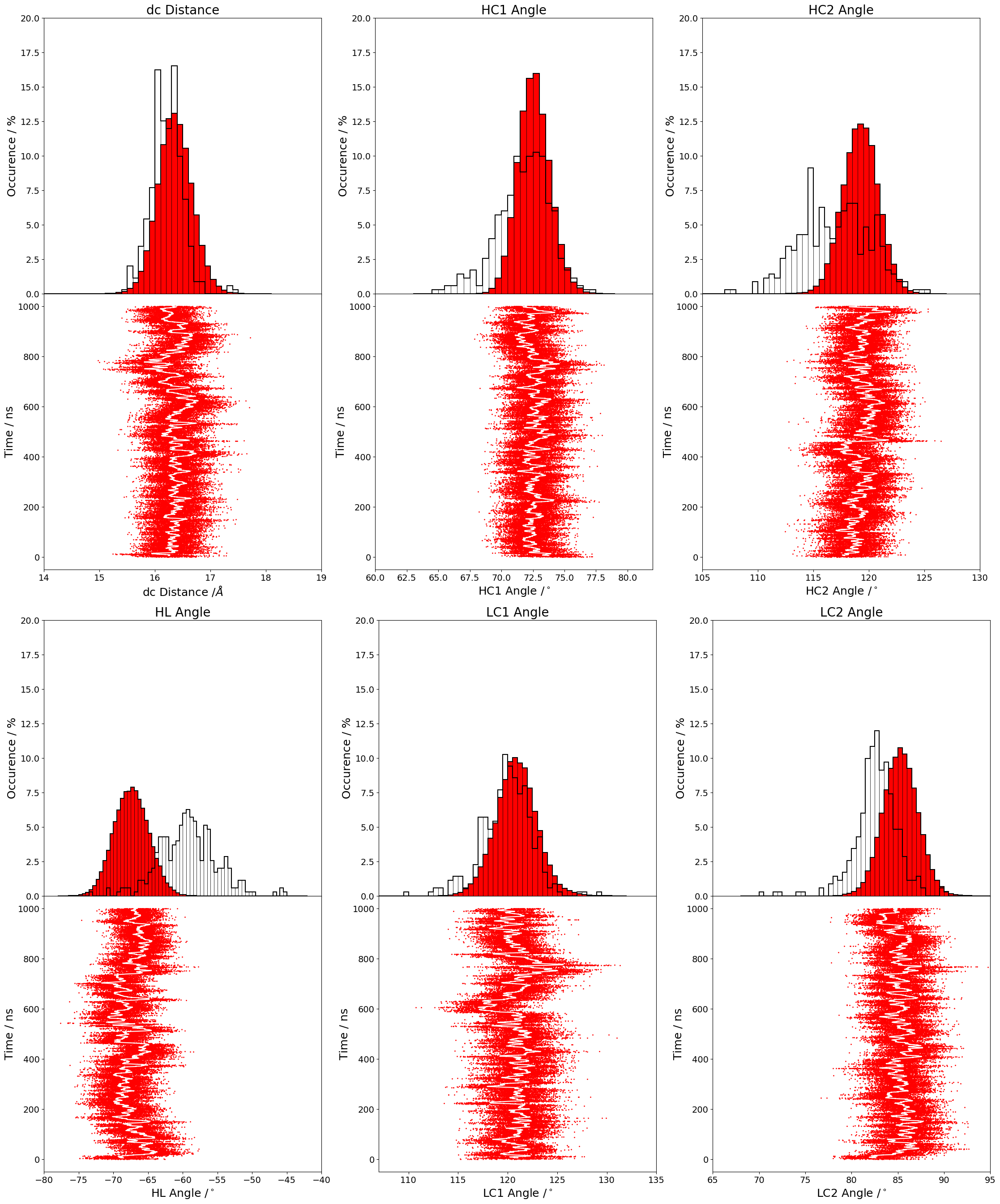


SI Figure S3: Complex scFv ABangle measure of 1 µs molecular dynamics simulation.


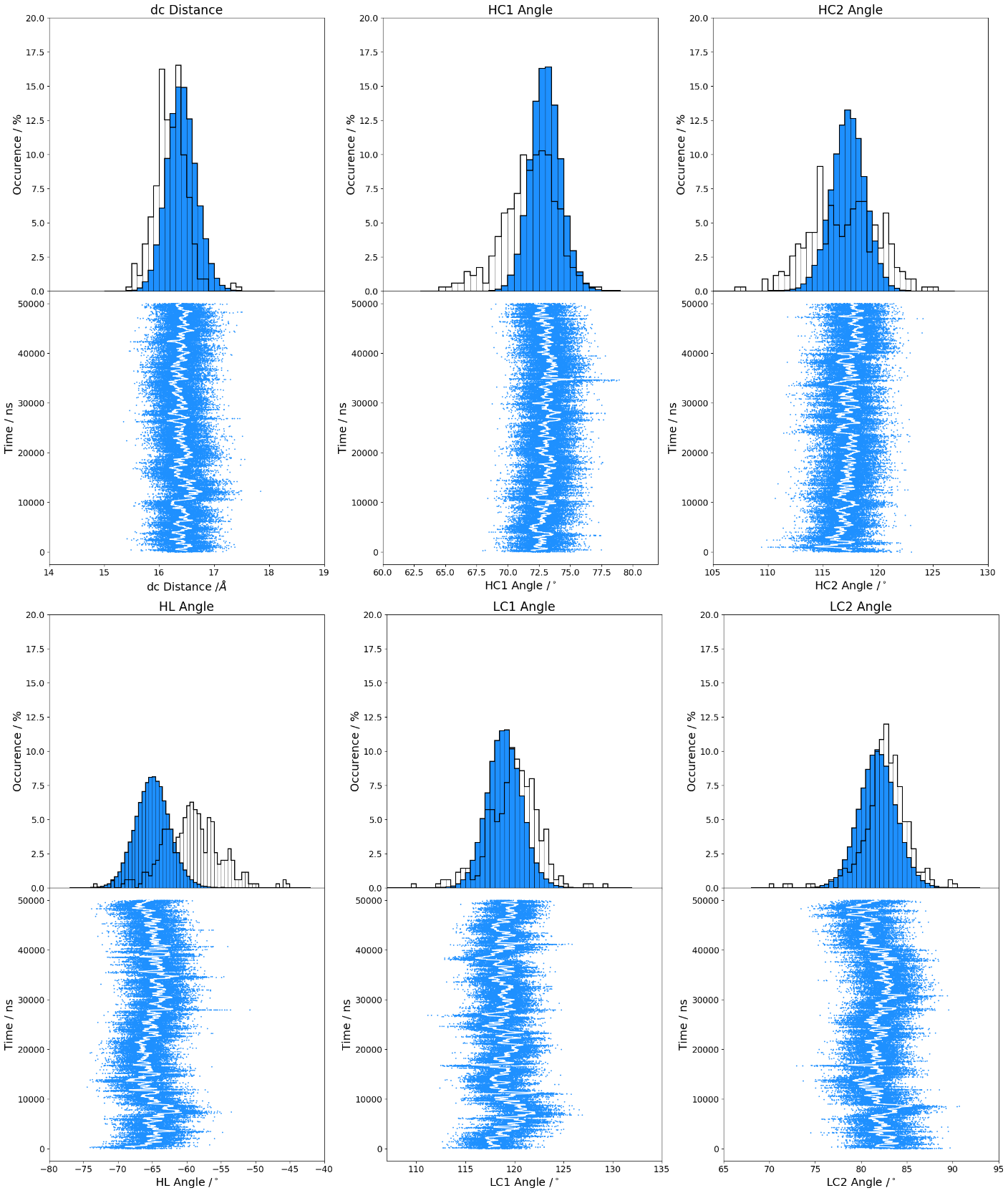
 SI Figure S4: Fab ABangle measures of 1 µs molecular dynamics simulation.


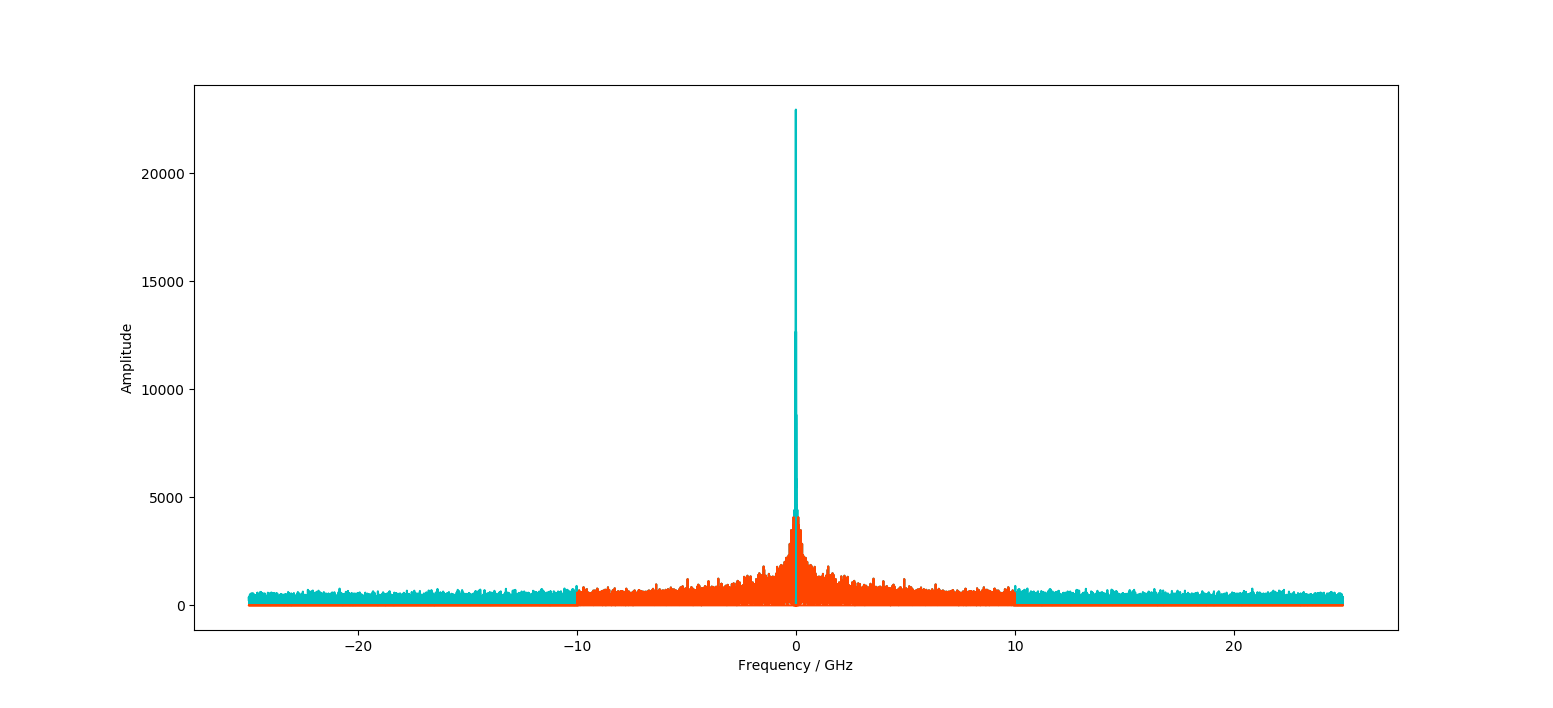


SI Figure S5: FFT Spectrum of the HL angle fluctuations of the Fab simulated NMR ensemble.


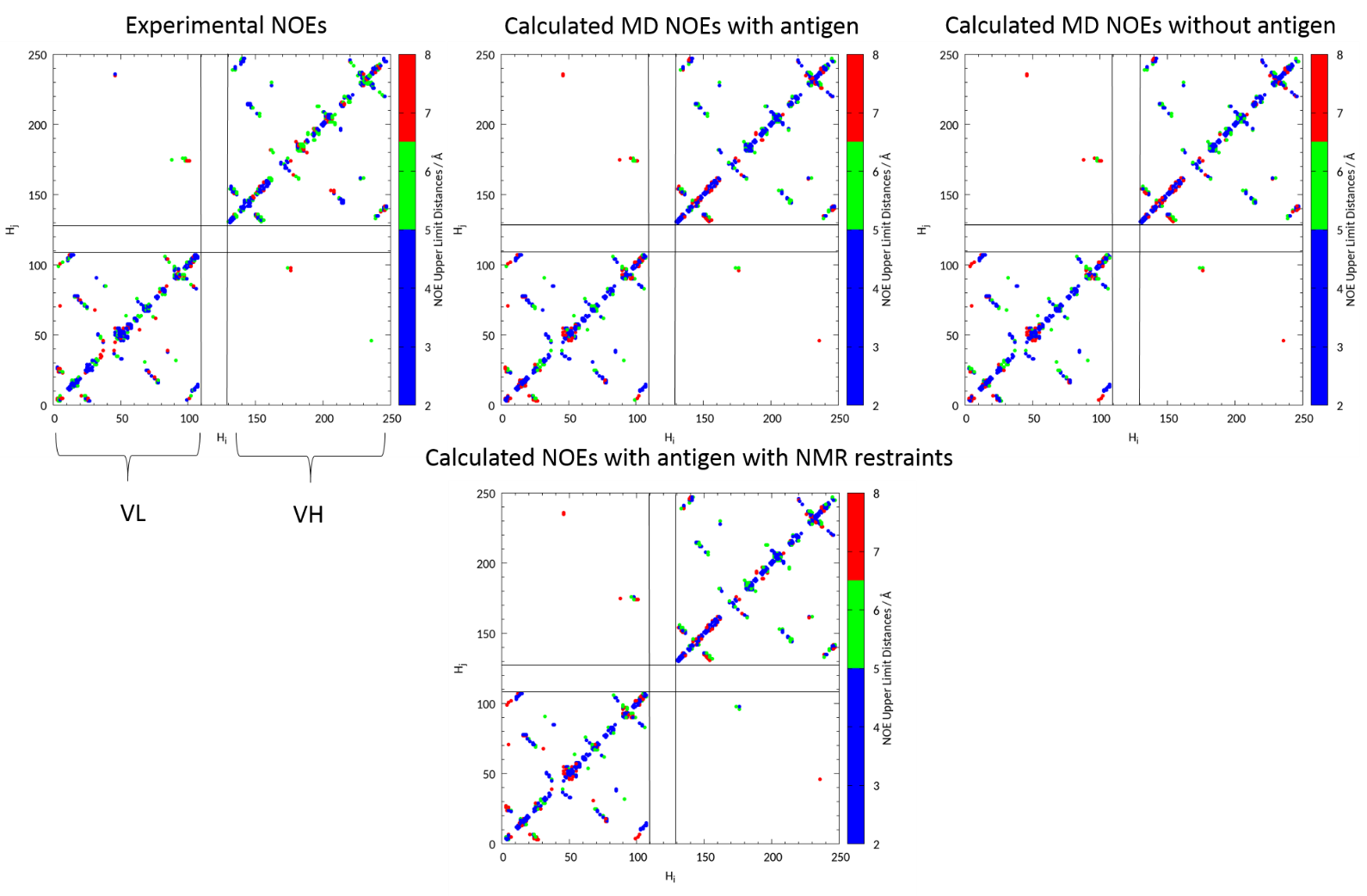


SI Figure S6: Comparison of experimental scFv complex NOEs with the calculated NOEs of 1 µs simulations with and without the antigen bound and with the complex NOE restraints. The plot shows the scFv fragment. The gap is caused by the peptide (G4S) linker, which directly connects the light and the heavy chain variable domains.


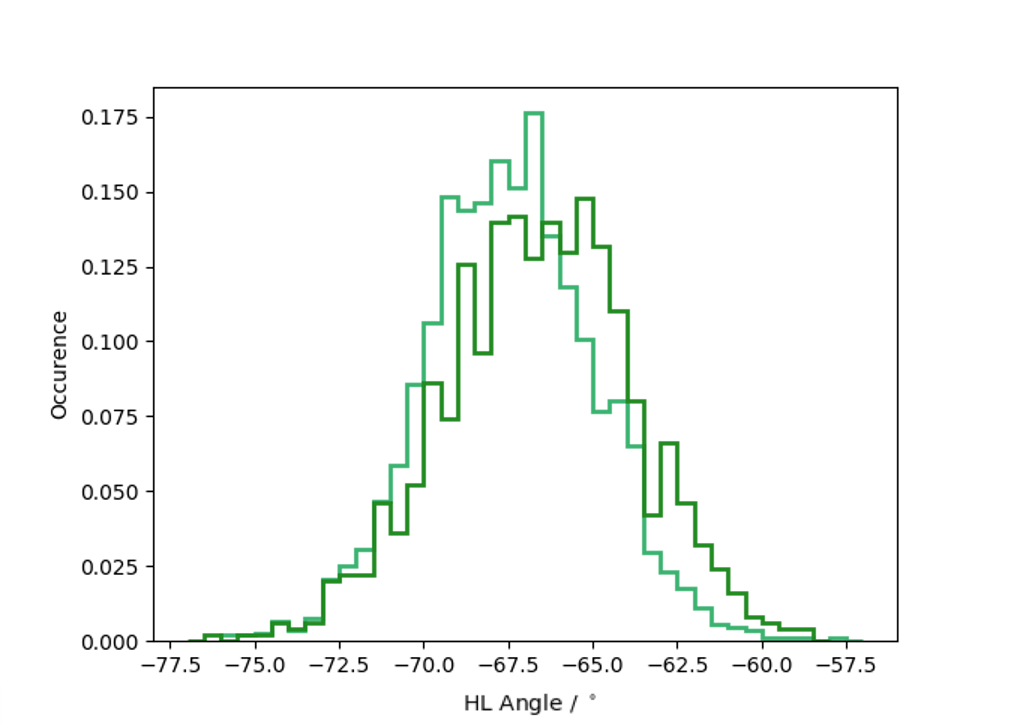


SI Figure S7: Overlay of the HL angle distributions of the Fv (green) and the scFv (forestgreen) fragments.


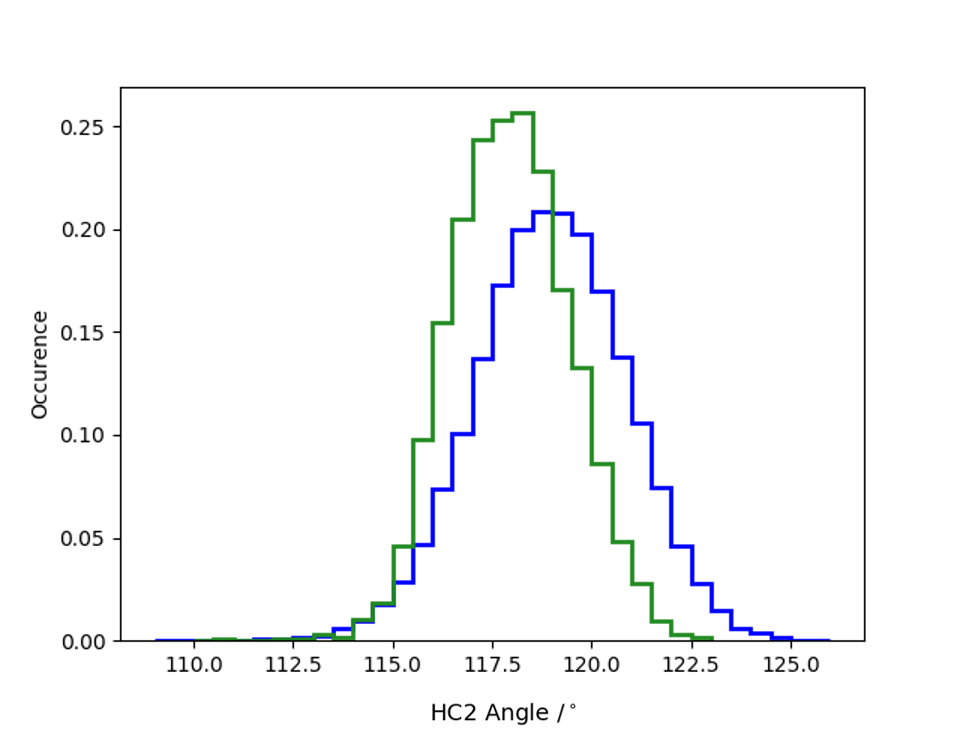


SI Figure S8: Overlay of the HC2 angle distributions of the Fv (green) and the NOE Fab simulations (blue) fragments

Table 1: Average and standard deviations of the six ABangle measures for all six considered antibody fragments.

| Antibody Fragments | HL / ° | HC1 / ° | LC1 / ° | HC2 / ° | LC2 / ° | dc / $\dot{A}$ |
| --- | --- | --- | --- | --- | --- | --- |
| Fv | -67.5$\pm$2.5 | 72.3$\pm$1.2 | 117.9$\pm$1.5 | 122.2$\pm$2.1 | 84.6$\pm$2.0 | 16.3$\pm$0.3 |
| scFv | -66.6$\pm$2.8 | 72.8$\pm$1.2 | 118.3$\pm$1.6 | 121.3$\pm$2.0 | 84.5$\pm$1.9 | 16.3$\pm$0.3 |
| complex | -67.4$\pm$2.5 | 72.6$\pm$1.2 | 119.2$\pm$1.6 | 120.8$\pm$2.0 | 85.2$\pm$1.9 | 16.4$\pm$0.3 |
| complex NMR | -65.7$\pm$3.4 | 72.2$\pm$1.9 | 120.9$\pm$2.3 | 122.6$\pm$2.4 | 82.5$\pm$2.3 | 16.5$\pm$0.3 |
| Fab | -65.0$\pm$3.1 | 73.0$\pm$1.2 | 117.3$\pm$1.5 | 119.0$\pm$1.7 | 81.8$\pm$2.0 | 16.4$\pm$0.3 |
| Fab NMR | -66.5$\pm$3.3 | 72.3$\pm$1.3 | 118.9$\pm$1.9 | 120.3 $\pm$1.8 | 83.0$\pm$2.1 | 16.4$\pm$0.3 |


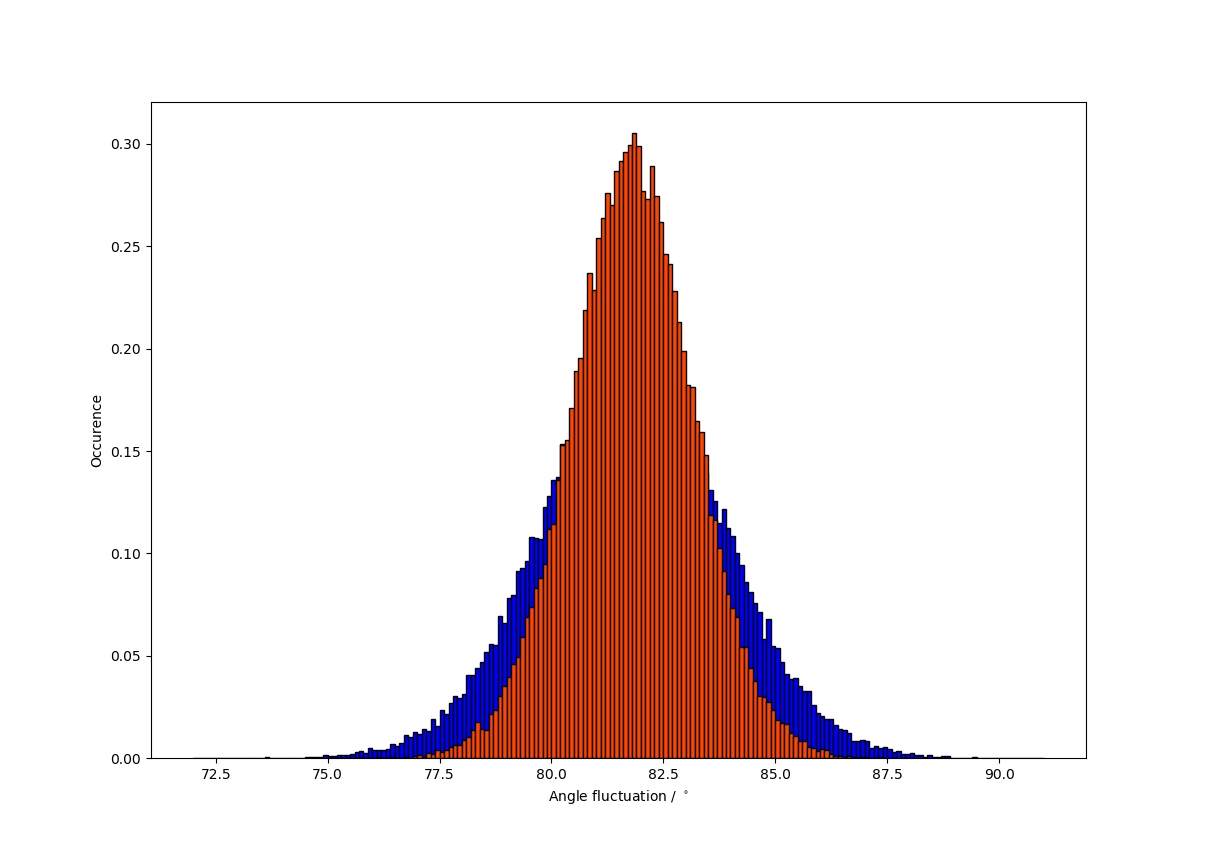


SI Figure S9: Overlay of the ABangle histogram (blue) with the angle variations observed in the 0.1 to 10 ns timescale (orange).


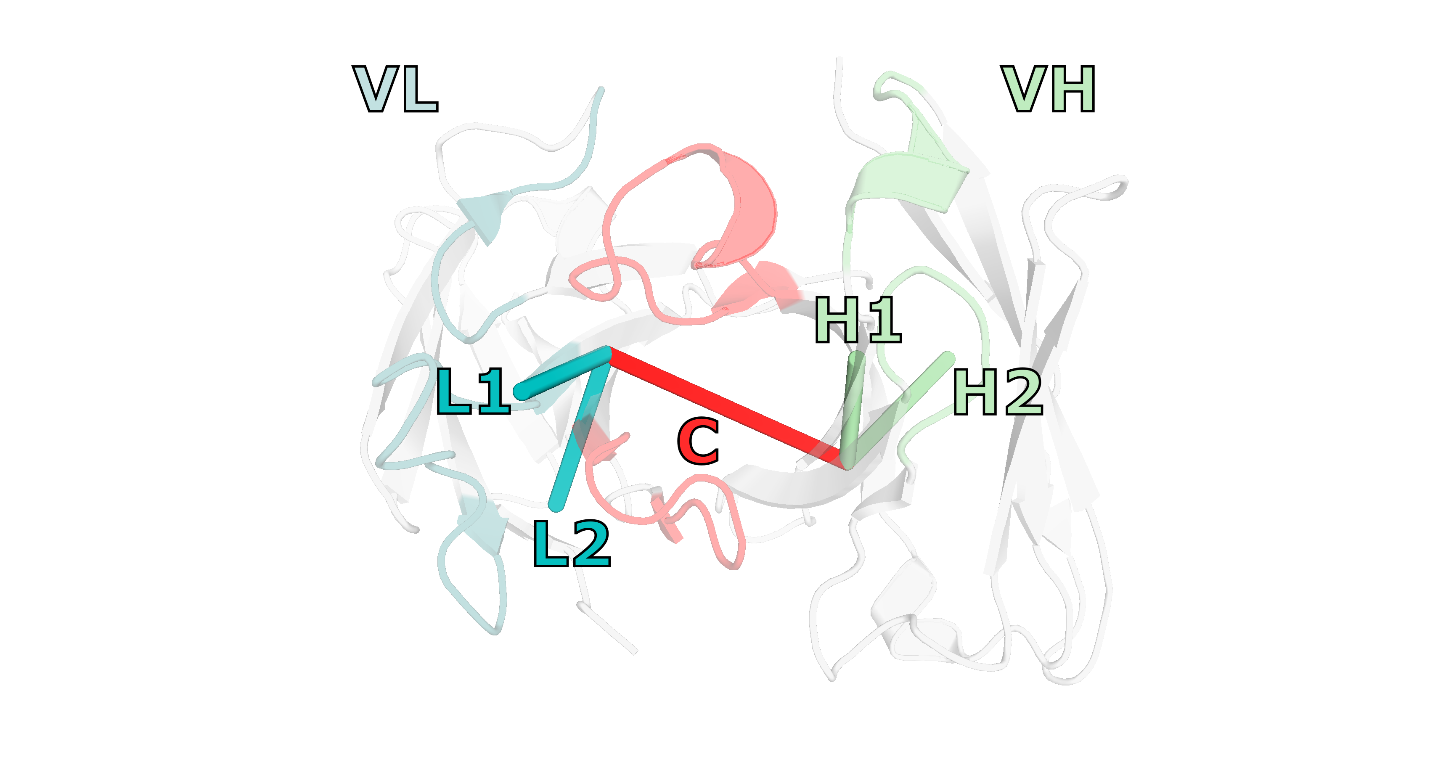


SI Figure S10: Illustration of the ABangle angle and distance definitions.
